# Interventions to support the re‐establishment of breastfeeding and their application in humanitarian settings: A systematic review

**DOI:** 10.1111/mcn.13440

**Published:** 2022-10-12

**Authors:** Nieves Amat Camacho, Johan von Schreeb, Francesco Della Corte, Ourania Kolokotroni

**Affiliations:** ^1^ Department of Global Public Health, Center for Research on Health Care in Disasters Karolinska Institute Stockholm Sweden; ^2^ Centre for Research and Training in Disaster Medicine,Humanitarian Aid, and Global Health Università del Piemonte Orientale Novara Italy; ^3^ Department of Nursing Cyprus University of Technology Limassol Cyprus

**Keywords:** artificial feeding, breast feeding, disasters, humanitarian, infant nutrition, re‐lactation

## Abstract

In 1998, the World Health Organisation (WHO) published general guidelines proposing essential measures to achieve relactation. Yet, increased knowledge about the practical set‐up of relactation support interventions in different contexts is needed, especially in humanitarian settings, where nonbreastfed infants are particularly at risk. This study aimed to compile and assess the characteristics, outcomes and factors influencing the implementation of relactation support interventions reported since the latest WHO recommendations. We conducted a systematic review following Preferred Reporting Items for Systematic Reviews and Meta‐analysis (PRISMA) guidelines, undertaking a search from Medline, Embase, PubMed Central, Web of Science, Global Health and CINAHL electronic databases. Studies published in English and Spanish, reporting characteristics and outcomes of relactation support provided to non‐(breastfeeding) BF mothers with infants aged less than 6 months were included. Data were analysed by narrative synthesis and the Johanna Briggs Institute Critical Appraisal Tools were used for quality assessment. Overall, 16 studies met the inclusion criteria. Most were observational and conducted in middle‐income countries, only one focused on humanitarian settings. Studies reported inpatient and community‐based interventions, which generally followed WHO recommendations for relactation. In 13 out of 16 studies, over 80% of mothers restarted BF after receiving relactation support. Enabling factors included younger infant age, shorter lactation gap, mother's strong motivation, family support, and continuous skilled support. Although current literature suggests that intensive relactation support can contribute to re‐establish BF, its application and effectiveness in humanitarian settings remain uncertain. Further research is needed to explore the effectiveness, feasibility and acceptability of different approaches to relactation support, especially in humanitarian settings.

## Key messages

- Providing intensive skilled support following the World Health Organisation's recommended measures for relactation can help mothers re‐establishing lactation, especially when combined with enabling factors (infant's young age, short lactation gap, and strong motivation).
- The feasibility and effectiveness of relactation support in humanitarian settings remain uncertain but are likely to be hampered during acute emergency phases and among populations with low baseline breastfeeding rates.
- Experimental studies are needed to explore the effectiveness of different approaches to relactation support. The feasibility and acceptability of relactation among different populations should be also assessed. Research in humanitarian settings should be prioritised.

## Graphical abstract

We reviewed studies (*n*= 16) reporting the characteristics, outcomes and implementation of relactation support interventions. Providing intensive skilled support can help mothers re‐establishing lactation, especially when combined with enabling factors (infant's young age, short lactation gap, and strong motivation). The feasibility and effectiveness of relactation support in humanitarian settings remain uncertain but are likely to be hampered during acute emergency phases and among populations with low baseline BF rates. More research is needed to explore the effectiveness of different approaches to relactation support among different population groups

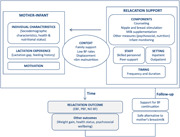

## 1 INTRODUCTION

The World Health Organisation (WHO) recommends exclusive breastfeeding (BF) for all infants up to 6 months of age (<6 m), and continued BF for at least until 2 years, according to the desire of the mother and child (WHO, 2003). It is estimated that scaling up BF practices almost universally could save the lives of around 820,000 children yearly, out of which 87% would be infants <6 m (Victora et al., 2016). In low‐ and middle‐income countries, nonbreastfed infants <6 m are particularly at risk and have been found to be 15 and 11 times more likely to die from pneumonia and diarrhoea, respectively, compared to those exclusively breastfed (Black et al., 2008).

Relactation is the process through which women who have ceased lactation can resume breastmilk production and start BF. In 1998, the WHO published a review of experiences and recommendations for the practice of relactation, including essential measures and factors influencing its achievement (WHO, 1998). However, these guidelines provide limited insights on the practical set‐up of relactation support interventions in different contexts (e.g., hospital or community‐based and humanitarian settings) or among specific groups, such as malnourished infants.

Humanitarian settings are those affected by natural or man‐made disasters causing widespread damage that exceeds the ability of the community to cope using its own resources (WHO, 2007). These settings are often characterised by a disruption of health services and result in forced population displacement, which negatively affects BF practice and support (Hirani et al., 2019; Rabbani et al., 2020). Still, BF is crucial for infants in these circumstances, since appropriate breastmilk substitutes may be inaccessible, and the risks of artificial feeding increase due to poor access to clean water, hygiene and health care (Arvelo et al., 2010; Haidar et al., 2017; Hipgrave et al., 2011). The Operational Guidance on Infant and Young Child Feeding in Emergencies recommends exploring the feasibility of relactation for all nonbreastfed infants in humanitarian settings and encourages the shift from mixed to exclusive BF, for those partially breastfed (IFE Core Group, 2017). However, the guidelines provide scarce operational and contextual considerations to achieve these goals. There is a recognised need to increase knowledge about the practical mechanisms contributing to effective relactation support (Burrell et al., 2020; Dall'Oglio et al., 2020; Prudhon et al., 2016).

The aim of this study is to describe the characteristics, related outcomes and associated factors of relactation support interventions reported since the publication of the WHO relactation guidelines in 1998. The findings will add updated operational perspectives and inform future recommendations for relactation support, with emphasis on its application in humanitarian settings.

The research questions, guiding this systematic review, are:

1. What were the characteristics of reported interventions supporting relactation among non‐BF mothers with infants <6 m, published after 1998?
2. What were the outcomes of those interventions?
3. What factors influenced the implementation and outcomes of those interventions?

## 2 METHODS

The methodology and reporting of this systematic review followed the Preferred Reporting Items for Systematic Reviews and Meta‐analysis (PRISMA) guidelines (Page et al., 2021). The review protocol was registered in the PROSPERO database and is accessible online (registration number CRD42020210043).

### 2.1 Search strategies and data sources

We conducted a literature search in Medline (Ovid), Embase, PubMed Central, Web of Science (Clarivate), Global Health (Ovid) and CINAHL (Ebsco) electronic databases, in September 2020, which was revised in September 2021. The keywords relact* and re‐lact* (a truncation of words for alternative endings) were used for the search, which was limited to studies published from 1998 until 2020 in English and Spanish languages. The search strategy was designed and performed in collaboration with experts from the library service at Karolinska Institutet (Supporting Information Material). We also conducted a grey literature search through Google Scholar, OpenGrey, the WHO Virtual Health Library (VHL) and Virtual Health Science Library (VHSL), UNICEF, and the Emergency Nutrition Network (ENN) web, using the terms ‘relactation’ and ‘re‐lactation’. The reference list of included studies was hand searched for relevant papers not identified in the primary search.

### 2.2 Eligibility criteria

We sought to identify studies that reported the implementation and outcomes of any intervention applied to support non‐BF mothers with infants aged 0−6 months to achieve relactation. Relactation was considered as the re‐establishment of BF in the biological mother of a child who was not BF, irrespective of the time of cessation at the beginning of the intervention. We did not consider studies in which relactation was attempted or undertaken by adoptive mothers or wet nurses, to specifically focus on the characteristics and outcomes of the predominant population group attempting relactation. When identified studies had a sample that comprised both biological and nonbiological mothers, as well as non‐BF and partially BF mothers, we included the study if the results were presented separately for each subgroup. The same principle was followed for studies including infants younger and older than 6 months (Table 1).

**Table 1:** Inclusion and exclusion criteria

Inclusion criteria	Exclusion criteria
Primary data/all study designs	Nonprimary data (e.g., reviews, opinions, books, and book chapter)
Population: biological mothers with infants aged 0−6 months who were not breastfeeding when recruited for the study and received support to achieve relactation	Studies focusing only on nonpuerperal induced lactation, wet nurses, mothers who are partially breastfeeding, and infants older than 6 months
Intervention: describing an intervention to support relactation and its outcomes	Not describing an intervention to achieve relactation or its outcomes
Published between 1998−2020 (included) in English or Spanish language	Outside the time and language limits

### 2.3 Study selection

**Figure 1:**
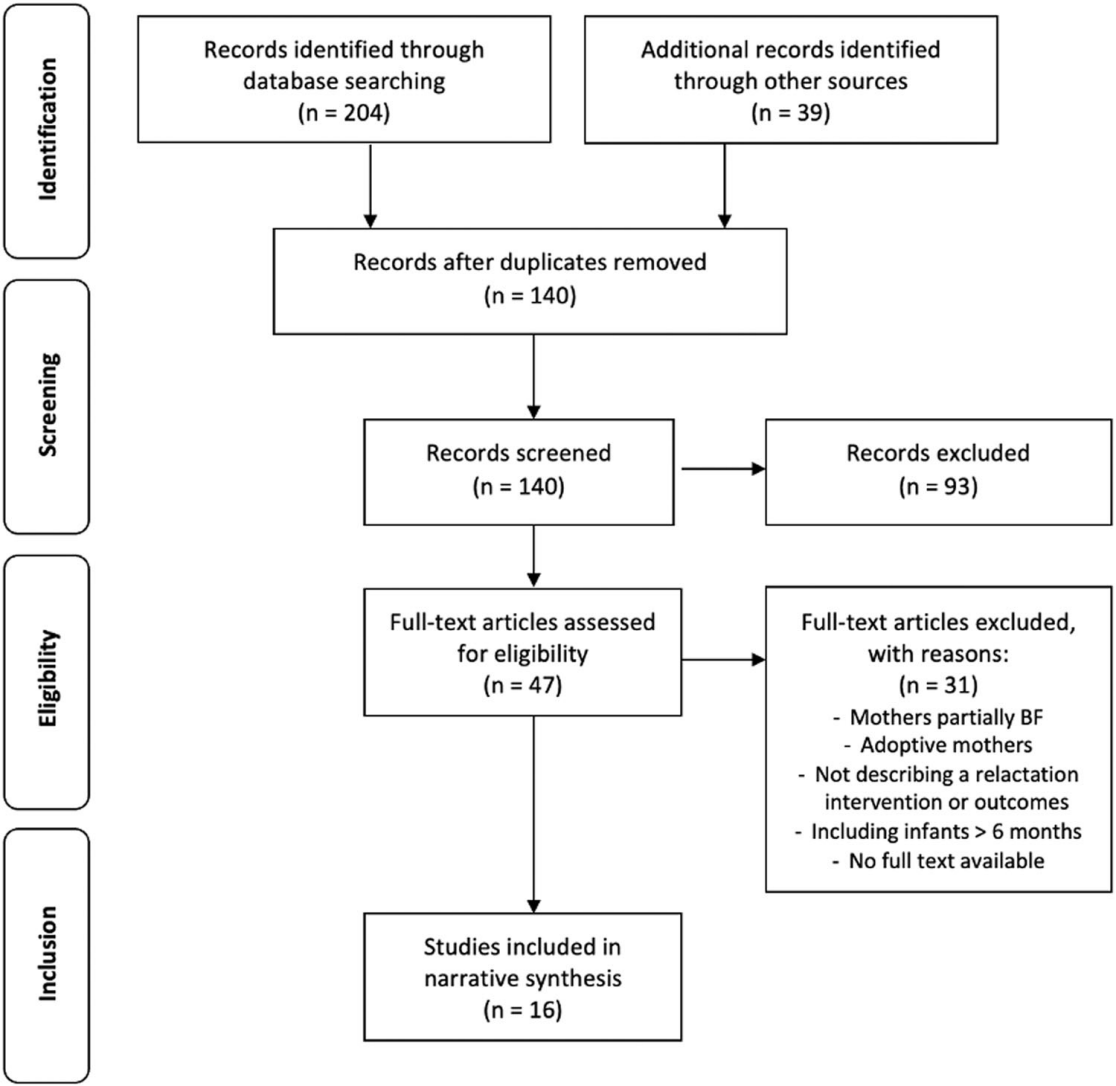
PRISMA flowchart for study selection. PRISMA, Preferred Reporting Items for Systematic Reviews and Meta‐analysis.

All results yielded through the search were imported into EndNote web. Duplicates were removed and all remaining titles and abstracts were screened for relevance. Those not relevant were excluded. Two researchers then read independently the full text of all relevant articles and assessed them against the eligibility criteria. Any discrepancies in the inclusion of studies were discussed until reaching a consensus (Figure 1).

### 2.4 Data extraction

The following data were extracted from each included study: title, author, year, publication type, country, study context, study design, objectives, study population, and characteristics, intervention characteristics, relactation outcome, other outcomes, time to achieve relactation, influencing factors, and recommendations. We categorised the possible relactation outcomes following an intervention as exclusive BF (i.e., feeding with breastmilk only without any other liquids or solids), mixed feeding (i.e., feeding with other liquids and/or foods and breastmilk) (WHO, 2016), or no relactation. These outcomes were presented as the number and percentage of women who achieved exclusive BF, mixed feeding, or no BF among those exposed to the intervention. We also recorded the measurements used to establish the BF status of mothers before intervention and the way relactation outcome was measured in each study. Other outcomes recorded were the time to achieve relactation, the infant's weight gain or nutritional status. All details describing the implementation of the intervention were collected, including the setting (i.e., outpatient or inpatient), type and duration of support, main components, resources used and follow‐up measures.

### 2.5 Data analysis

Given the quality and characteristics of the available studies we considered narrative synthesis was the most appropriate method for data analysis, for which we followed the guidelines by Popay et al. (2006) First, we drafted a framework following the structure of the WHO relactation guidelines, containing sections where the emerging data could be enclosed. We then performed a preliminary synthesis and explored data relationships within and between studies to gather the final results.

To analyse the quality of included studies we used the Johanna Briggs Institute (JBI) Critical Appraisal Tools for systematic reviews, concretely the assessment forms proposed for case series, case reports, cross‐sectional, cohort (longitudinal) and quasi‐experimental studies. (Johanna Briggs Institute, University of Adelaide, n.d.) The score of each study was determined by giving its adherence to the items listed in the forms—considered as yes/no/unclear/nonapplicable. The number of positively valued items over the total was measured for each study and type of study. Acknowledging the heterogeneity and the limited number of studies available informing on this subject, we decided not to exclude any study based on its quality.

Since this review used secondary data already published in other studies, no ethical approval was sought.

## 3 RESULTS

In total, 16 studies describing the characteristics and outcomes of relactation support interventions met the inclusion criteria and were considered for analysis. The characteristics of the included studies are presented in Table 2.

**Table 2:** Characteristics of included studies (*n* = 16)

Author, year	Country	Publication type	Study design	Score JBI quality assessment
Abul‐Fadl et al. (2012)	Egypt	Journal article	Longitudinal	7/10
Agarwal & Jain (2010)	India	Letter to editor	Case report	5/7
J. G. Alves et al. (1999)	Brazil	Brief report	Longitudinal	5/10
Banapurmath et al. (2003)	India	Brief report	Descriptive	4/10
Burrell et al. (2020)	Bangladesh	Journal article	Descriptive	7/10
Cluet de Rodriguez et al. (2014)	Venezuela	Journal article	Longitudinal	10/10
De et al. (2002)	India	Brief report	Descriptive	6/10
Fuenmayor et al. (2004)	Venezuela	Journal article	Longitudinal	5/10
Gallardo (2017)	Philippines	Conference abstract	Descriptive	2/10
Kayhan‐Tetik et al. (2013)	Turkey	Case report	Case report	6/7
Mehta et al. (2018)	India	Journal article	Cross‐sectional	6/8
Menon & Mathews (2002)	India	Letter to editor	Case report	4/7
Muresan (2011)	Romania	Case report	Case report	7/7
Nuzhat et al. (2019)	Bangladesh	Journal article	Retrospective longitudinal	6/11
Nyati et al. (2014)	South Africa	Journal article	Quasi‐experimental	7/8
Tomar (2016)	India	Journal article	Descriptive	5/10

Abbreviation: JBI, Johanna Briggs Institute.

All included studies were observational, except for one (Nyati et al., 2014). In terms of quality, the mean number of items positively valued in descriptive (*n* = 5), case report (*n* = 4), cross‐sectional (*n* = 1), longitudinal (*n* = 5) and quasi‐experimental studies (*n* = 1) was 4.8/10, 5.5/10, 6/8, 6.6/10 and 7/8, respectively (Supporting Information Material). Most descriptive studies (*n* = 4) failed to provide clear information about the study site or the consecutive and complete inclusion of subjects (Banapurmath et al., 2003; De et al., 2002; Gallardo, 2017; Tomar, 2016) and none of them stratified results by participants' clinical or nutritional conditions. In two studies, the achievement of relactation was not the primary outcome (J. G. Alves et al., 1999; Cluet de Rodríguez et al., 2014). Only one study (Nuzhat et al., 2019) described the method for measuring BF status before the intervention and the relactation outcome at the end, in this case using WHO 24‐hour recall method. Other authors (*n* = 5) mentioned the need and quantity of top feeds as the way to assess whether full or partial relactation was achieved (Agarwal & Jain, 2010; De et al., 2002; Mehta et al., 2018; Nyati et al., 2014; Tomar, 2016). The length of time that exclusive BF or mixed feeding was sustained was only described in three studies (Kayhan‐Tetik et al., 2013; Muresan, 2011; Nyati et al., 2014).

### 3.1 Intervention characteristics

The main components of the interventions reported are summarised in Table 3. Most interventions (*n* = 11) targeted infants hospitalised with diverse pathologies (predominantly diarrhoea, respiratory infections, dehydration, and malnutrition) (Agarwal & Jain, 2010; J. G. Alves et al., 1999; Cluet de Rodríguez et al., 2014; De et al., 2002; Fuenmayor et al., 2004; Gallardo, 2017; Kayhan‐Tetik et al., 2013; Mehta et al., 2018; Menon & Mathews, 2002; Nuzhat et al., 2019; Tomar, 2016), some of those directly associated with lactation failure (Kayhan‐Tetik et al., 2013; Tomar, 2016). One included HIV‐positive infants (Nyati et al., 2014) and four targeted healthy nonbreastfed infants (Abul‐Fadl et al., 2012; Banapurmath et al., 2003; Burrell et al., 2020; Muresan, 2011). All interventions, except for two outpatient‐based (Abul‐Fadl et al., 2012; Nyati et al., 2014), provided daily support during the time of hospital admission or the first days of relactation. The number of times and duration of support in each encounter were only reported in three studies (Banapurmath et al., 2003; Menon & Mathews, 2002; Nuzhat et al., 2019). Three interventions involved specialised BF or lactation management units (De et al., 2002; Gallardo, 2017; Nuzhat et al., 2019), and nine provided support by staff specifically trained. In six interventions, the husband or the family was directly involved (Agarwal & Jain, 2010; Banapurmath et al., 2003; Burrell et al., 2020; De et al., 2002; Nuzhat et al., 2019; Tomar, 2016).

**Table 3:** Reported characteristics of reviewed relactation support interventions

Author, year	Counselling	Specialised staff	Breast stimulation	Milk supplementation	Infant monitoring	Duration and follow‐ up	Others
Abul‐Fadl et al. (2012)	Individual	Yes	24 h room‐in, skin‐to‐skin contact, frequent nipple stimulation	‐	Weight	Phone follow‐up biweekly for 6−8 weeks	Locally available herbal lactogogue
							Use 3 different communication approaches
Agarwal & Jain (2010)	Individual + relatives	Yes	Supplementary suckling with tube + syringe	Cup‐spoon Tube + syringe	Weight	2 weeks	Provision of comfortable environment, back massage
J. G. Alves et al. (1999)	Individual	‐	Frequent suckling, Lact‐Aid supplementer	Lact‐Aid supplementer	Weight	During hospitalisation (mean = 6.9 days)	
Banapurmath et al. (2003)	Individual + relatives	Yes	Frequent suckling, skin‐to‐skin contact, bedding‐in, manual breastmilk expression, drop and drip technique	Cup‐spoon	Weight + urine output	10 days, up to 3−4 h/day	Constant self‐confidence building and positive reinforcement provided. Telephone service available if doubts or anxiety
Burrell et al. (2020)	Individual + relatives	Yes	Supplementary suckling technique	‐	Weight	Daily household visits until confidence was built	
Cluet de Rodriguez et al. (2014)	Individual	‐	Frequent suckling, drop and drip technique, hand and mechanical expression	‐	‐	‐	
De et al. (2002)	Individual + relatives	Yes	Frequent suckling, bedding‐in, skin‐to‐skin contact, drop and drip technique	Infant formula Cup‐spoon	Weight	During hospitalisation (maximum 3 weeks). Follow‐up till 9 months of age	Adequate food and rest along with constant motivation
Fuenmayor et al. (2004)	Individual	‐	Supplementary suckling technique	‐	Weight	During hospitalisation (8 days). Follow‐up every month for 3 months	
Gallardo (2017)	Individual	Yes	Breast massage, hand expression, drop and drip technique	Cup feeding	Weight	During hospitalisation, then weekly household visits up to 21 days	Lecture about BF
Kayhan‐Tetik et al. (2013)	Individual	Yes	Breast massage, supplementary suckling with tube + syringe	Artificial milk Tube + syringe	Weight + urine output	6 days	Provision of constant motivation and support to build up mother's self‐confidence
Mehta et al. (2018)	Group + individual	Yes	Frequent suckling, milk expression and drop and drip technique	Animal milk Cup feeding after every suckling time	Weight + health status	During hospitalisation, follow‐up for 4 months	Adequate nutrition and rest, supplementation of iron, folic acid, calcium for the mother. Lactogogue and placebo
Menon and Mathews (2002)	Individual	‐	Stimulate suckling and manual breastmilk expression	Expressed breastmilk, through nasogastric tube in 1 case	Weight	Support every 2 h during hospitalisation	
Muresan (2011)	Individual	Yes	Breast massage, manual and mechanical milk expression, skin‐to skin‐contact, bedding‐in and supplementary suckling technique	Infant formula or aunt's breastmilk BF supplementer or bottle	Weight	Household visits for 5 weeks	Lactogogues (domperidone + herbal preparation)
Nuzhat et al. (2019)	Individual + relatives	Yes	‐	‐	‐	2 to 3 times/day during hospitalisation	Mental health support for building up mothers' confidence.
Nyati et al. (2014)	Individual	Yes	‐	‐	Growth + health status	Weekly over 4 weeks and then less frequently until 24 weeks	2‐day workshop with standard curricula
Tomar (2016)	Individual + relatives	Yes	Frequent suckling, supplementary suckling or drop and drip technique	*Paladai* or cup‐spoon after every suckling time	Weight	During hospitalisation, then household follow‐up	Rest, good hygiene and diet for the mother, psychological support if needed.

All studies, except the one by Gallardo (2017), reported an initial assessment of mothers and infants. All provided encouragement and practical information about BF and the relactation process. Two added lectures or training workshops (Gallardo, 2017; Nyati et al., 2014). Three interventions provided group as well as individual counselling (Agarwal & Jain, 2010; Mehta et al., 2018; Nuzhat et al., 2019).

For breast stimulation, frequent suckling, hand or mechanical milk expression were recommended and supported, as well as the use of supplementary suckling technique (J. G. Alves et al., 1999; Burrell et al., 2020; Fuenmayor et al., 2004; Kayhan‐Tetik et al., 2013; Muresan, 2011; Tomar, 2016) or drop and drip technique (Banapurmath et al., 2003; Cluet de Rodríguez et al., 2014; De et al., 2002; Gallardo, 2017; Mehta et al., 2018; Tomar, 2016). Two studies used a supplementary suckling technique by pouring the milk over the nipple through a tube connected to a syringe (Agarwal & Jain, 2010; Kayhan‐Tetik et al., 2013). Frequent skin‐to‐skin contact, bedding‐in and the withdrawal of pacifiers and bottles to avoid nipple confusion were also encouraged. Lactogogues were prescribed in three interventions (Abul‐Fadl et al., 2012; Mehta et al., 2018; Muresan, 2011). Six interventions encouraged cup or cup‐spoon feeding to provide top milk (Agarwal & Jain, 2010; De et al., 2002; Gallardo, 2017; Mehta et al., 2018; Tomar, 2016). Studies generally presented scarce information about how milk supplementation was carried out, in terms of the milk used (e.g., expressed breastmilk and artificial milk), its preparation and the quantities needed to give and reduce as the milk flow increased.

In three interventions mothers received targeted mental health support to build up their confidence (De et al., 2002; Nuzhat et al., 2019; Tomar, 2016). Other interventions (*n* = 4) provided or encouraged good nutrition and rest for the mothers (Agarwal & Jain, 2010; De et al., 2002; Mehta et al., 2018; Tomar, 2016). Mehta et al. (2018) advised micronutrient supplementation for the duration of the study. Over 80% of studies reported measuring infant's weight daily or regularly, and some (*n* = 4) also monitored other parameters, such as urine output or infants' general health status. Nyati et al. (2014) also measured the mother's weight at each visit.

### 3.2 Intervention outcomes

#### 3.2.1 Percentage of women achieving relactation

The percentage of women achieving relactation varied between studies, ranging from 10% to 100%. Summarising all mother−infant pairs targeted in the included studies (*n* = 2478), 79,5% (*n* = 1972) mother‐infant dyads restarted lactation. Around half (48.5%) achieved exclusive BF (*n* = 1202) and 28.3% provided mixed feeding (*n* = 703) after the intervention. The achievement of exclusive BF was high following the interventions reported in case studies and the studies by Banapurmath et al. (2003), De et al. (2002), Gallardo (2017), Mehta et al. (2018) and Tomar (2016). However, the attainment of exclusive BF was limited in other studies (Abul‐Fadl et al., 2012; J. G. Alves et al., 1999; Burrell et al., 2020; Nuzhat et al., 2019) (Table 4).

**Table 4:** Setting, target population and outcomes of reviewed relactation support interventions

Author, year	Setting	Study population	Lactation gap	Relactation outcome	Days to achieve relactation
Abul‐Fadl et al. (2012)	Outpatient *Maternal and child welfare centres* + *phone support*	200 healthy mothers with healthy infants aged < 3 months	Not specified	10% (20/200) achieved mixed feeding	Not specified
Agarwal & Jain (2010)	Inpatient *Hospital*	1 mother with her infant aged 14 weeks admitted with severe dehydration	Since birth (14 weeks)	1/1 achieved exclusive BF	Few drops of milk were secreted by Day 6, exclusive BF by Day 11
J. G. Alves et al. (1999)	Inpatient *Maternal and Child Institute hospital*	163 mother and infants aged between 30 and 90 days hospitalised due to diarrhoea or pneumonia	At least 2 weeks	27,61% (45/163) achieved exclusive BF	Not specified
Banapurmath et al. (2003)	Outpatient *Paediatric outpatient clinic*	916 healthy mothers with their healthy infants aged < 6 weeks	From 1 to 45 days	91,6% (839/916) achieved relactation (83.4% exclusive BF; 8.2% mixed feeding)	Mixed feeding: 2−5 days in babies with lactation gap < 2 weeks, 5−10 days in babies with lactation gap > 2 weeks.
					Exclusive BF: 3−10 days in the first group, not be achieved in the second group
Burrell et al. (2020)	Outpatient *Infant and Young Child Feeding programmes in displacement camps and settlements. Save The Children (NGO)*	2 mothers with infants aged < 6 months	Not specified	1/2 mothers achieved relactation (unspecified if exclusive BF)	Not specified
Cluet de Rodriguez et al., 2014	Inpatient *Emergency Paediatric Service at University hospital*	15 mothers with infants aged < 6 months hospitalised for different pathologies	At least 2 weeks	100% (15/15) achieved relactation (unspecified if exclusive BF)	The mean time of occurrence of milk secretion was 6 + 1.60 days (range between 4 and 9)
De et al. (2002)	Inpatient + Outpatient follow‐up *Lactation Management Unit at Nutrition Rehabilitation Centre. Child In Need Institute (NGO)*	137 mothers with their infants aged < 6 months hospitalised with acute respiratory infections or diarrhoea and malnutrition	At least 10 days	84% (115/137) achieved relactation (60.5% exclusive BF; 21.8% mixed feeding)	Complete relactation took an average of 15−20 days, the maximum being 40 days for one mother
Fuenmayor et al. (2004)	Inpatient + Outpatient follow‐up Emergency Paediatric Service at University hospital	50 mothers and infants aged 2−5 months, hospitalised for diverse pathologies	At least 1 month	100% (50/50) achieved relactation (unspecified if exclusive BF)	The average beginning of the production of milk was of 6.6 ± 2.4 days
Gallardo (2017)	Inpatient + Outpatient follow‐up	35 mothers of sick infants aged 0−6 months	Not specified	97.1% (34/35) achieved relactation (71.4% exclusive BF; 25.7% mixed feeding)	Not specified
	*Lactation Management Team at National Children Hospital*				
Kayhan‐Tetik et al. (2013)	Inpatient *Hospital*	1 mother and her 8‐week‐old infant hospitalised for acute gastroenteritis and dehydration	Never breastfed	1/1 achieved exclusive BF and sustained for 4 months	First milk secretion at Day 3. Exclusive BF achieved on Day 6
Mehta et al. (2018)	Inpatient + Outpatient follow‐up *Tertiary care referral hospital*	33 mothers and their hospitalised infants < 4 months	2 weeks	100% (33/33) achieved relactation (63.6% exclusive BF; 36.3% mixed feeding)	Bottle‐fed infants: mixed feeding = mean 18 days; exclusive BF = mean 29.1 days
					Cup‐feeding infants: mixed feeding = mean 9.7 days; exclusive BF = mean 21 days
Menon & Mathews (2002)	Inpatient *Hospital*	3 high‐risk infants (one preterm baby, two infants with Pierre‐Robin syndrome) aged between 2 and 7 weeks	Never breastfed	3/3 mothers achieved relactation (2 exclusive BF; 1 mixed feeding)	4 days to start secreting milk
Muresan (2011)	Outpatient *Homebased*	1 mother and her healthy infant aged 8 weeks	At least 6 weeks	Achieved exclusive BF and sustained up to 7 months	Colostrum appeared 4 days after relactation started, exclusive BF achieved after 30 days
Nuzhat et al. (2019)	Inpatient *BF Counselling Unit at hospital*	531 infants aged < 6 months hospitalised for diarrhoeal illness	Not specified	90% (478/531) achieved relactation (4.3% exclusive BF; 85.7% mixed feeding)	Not specified
Nyati et al. (2014)	*Outpatient Perinatal HIV Research Unit at hospital outpatient clinic*	9 mothers of HIV infected infants aged < 12 weeks of age	7/9 never breastfed	100% (9/9) achieved relactation (3/9 exclusive BF sustained for 24 weeks; 7/9 mixed feeding)	Exclusive BF achieved from 7 to 21 days
Tomar (2016)	Inpatient + Outpatient follow‐up *Army hospital*	381 mothers with infants aged < 6 months hospitalised for various illnesses or malnutrition.	From 7 days to 3 months	85.8% (327/381) achieved relactation (61.1% exclusive BF; 24.6% mixed feeding)	3−18 days to initiate lactation, 5−28 days to establish lactation

Abbreviations: BF, Breastfeeding; NGO, Non‐Governmental Organisation.

#### 3.2.2 Time to achieve relactation

The time required for mothers to start producing milk and establish BF was presented in 11 studies. Among mothers who relactated, the start of milk secretion varied from 2 days up to 15 days. Mixed feeding was achieved between 2 and 18 days, and the establishment of exclusive BF took from three up to 30 days (Table 4).

#### 3.2.3 Other outcomes

Positive weight gain and growth were reported among infants of mothers who achieved relactation (De et al., 2002; Fuenmayor et al., 2004; Kayhan‐Tetik et al., 2013; Menon & Mathews, 2002; Muresan, 2011). In one study, weight gain among infants who relactated was higher than among those who did not (*p* < 0.001). Those achieving relactation also had a lower incidence of hospital‐induced malnutrition (risk ratio = 0.30; 95% confidence interval [0.15−0.61]) (J. G. Alves et al., 1999). Likewise, De et al. (2002) found that the episodes of illnesses decreased among infants who relactated compared to those who did not. According to Nyati et al. (2014) no statistically significant differences were seen in BF groups and non‐BF groups in terms of infants' growth, CD4 count and reported sick visits at the end of the study. One study evaluated the changes in behaviours encouraged to facilitate relactation (e.g., stop the use of pacifiers and bottles), all of which improved following the intervention (Abul‐Fadl et al., 2012). Two studies reported an increase in mother's satisfaction and self‐confidence following the achievement of relactation (Kayhan‐Tetik et al., 2013; Nyati et al., 2014), and another suggested an improvement in mother−infant psycho‐affective relationship (Fuenmayor et al., 2004). Negative attitudes among the mothers who did not achieve relactation were also noted in one case, as they felt discouraged when willing and trying to breastfeed their infants and not managing to do so (Nyati et al., 2014).

### 3.3 Factors affecting the implementation and outcomes of interventions

#### 3.3.1 Related to the infant

Younger age of the infant was associated with a higher chance to achieve relactation (Banapurmath et al., 2003; De et al., 2002; Mehta et al., 2018; Tomar, 2016). One study found that mothers with infants less than 6 weeks were more likely to achieve exclusive BF (*p* < 0.001) (Mehta et al., 2018). In the study by Tomar (2016) over 81% of infants aged less than 2 months achieved exclusive BF, in comparison with only 24% of infants older than 4 months.

#### 3.3.2 Related to the mother

One intervention found mothers less than 25 years more likely to achieve full relactation (*p* < 0.05) (Mehta et al., 2018). In the study by Abul‐Fadl et al. (2012) all the mothers who achieved relactation were educated, compared to those who did not achieve it, who were mainly illiterate. A strong motivation of mothers was subjectively perceived to be the most important enabler for the achievement of relactation in most studies. Mehta et al. (2018) also hypothesised that the motivation of mothers in their study was high because their infants were sick. Another identified success factor was the capacity of the intervention to bring back the mother's self‐confidence (De et al., 2002). However, mothers' BF self‐efficacy was not systematically assessed in any reviewed study.

#### 3.3.3 Related to previous lactation practice

The feeding practices before trying relactation influenced its success. Better results were obtained when there was a shorter lactation gap (i.e., time since discontinuation of BF) (Banapurmath et al., 2003; De et al., 2002; Mehta et al., 2018; Tomar, 2016). Infants on bottle‐feeding also took a longer time to initiate relactation and presented more difficulties due to nipple confusion (De et al., 2002; Mehta et al., 2018; Tomar, 2016). None of the reviewed studies looked at a mother's BF experience with previous children and its possible effect on relactation outcomes. Only Muresan (2011) described that the mother supported in their case had breastfed her previous child for 18 months.

#### 3.3.4 Related to intervention design and implementation

Studies (*n* = 4) described barriers to engaging mothers in relactation support interventions. In the study targeting HIV‐positive mothers, two‐thirds of eligible mothers declined outpatient support for relactation, due to work commitments, not wishing to BF or being reluctant to BF thinking they may ‘poison’ their infants (Nyati et al., 2014). During the Rohingya humanitarian crisis, among 15 nonbreastfed infants identified, only two mothers tried relactation (Burrell et al., 2020). In another study, 75% of enroled mothers preferred to be followed up only by phone calls, instead of meeting an available lactation specialist in an outpatient clinic, which they considered to be too far to reach (Abul‐Fadl et al., 2012). In the same line, Banapurmath et al. (2003) reported that women accepted outpatient follow‐up more easily than inpatient admission, exposing financial constraints, lack of family support and other recent admissions to the hospital. Abul‐Fadl et al. (2012) assumed that recruiting mothers from health centres where they attended to receive supplies of subsidised infant formula interfered with the success of their intervention.

Inpatient support was considered to have several advantages over outpatient: the constant encouragement and guidance by skilled staff, and more comprehensive support to the mother including appropriate nutrition, rest and protection from the stress at home. At inpatient settings mothers also had access to other women in the same process of relactation, and infants were better followed up. However, the inpatient intervention by Nuzhat et al. (2019) was hardly effective to achieve exclusive BF, which the authors linked to the fact that support was only provided during the hospital stay. This was frequently a short period and mothers were not followed up after discharge. The same issue may have contributed to the little effect of the intervention run by J. G. Alves et al. (1999).

In outpatient settings, a multifaceted package of intensive support applied for 10 days resulted in high rates of relactation and exclusive BF (Banapurmath et al., 2003). Notably, this study included very young infants with a short lactation gap and excluded sick and preterm infants and mothers with breast problems, which could have contributed to the high impact of the intervention. Muresan (2011) reported encountering challenges hindering the achievement of relactation (e.g., infant's refusal of the breast and supplementary suckling technique device, slow weight gain) that finally could be overcome thanks to intensive home‐based support. Burrell et al. (2020) choose to provide outpatient support to nonbreastfed infants during the Rohingya crisis as only a few of them were identified and the set‐up of a dedicated inpatient facility for relactation was not justified. Several challenges were faced with this approach, those related to limited guidance about milk supplementation, lack of family support at home, limited skilled staff to conduct household visits, difficulties to maintain the mother's motivation, and poor hygiene practices for the care of supplementary suckling technique equipment.

Some feeding practices that were encouraged during relactation support likely contributed to the achievement of exclusive BF. These include stopping the use of bottles and pacifiers and shifting to cup or cup‐spoon feeding; nonrestricted access of the infant to the breast (including nighttime feeding); longer suckling time and correct positioning and latching during feeding (Abul‐Fadl et al., 2012; Nyati et al., 2014). Creating a comfortable space for the mother and choosing a supplementary suckling technique that was accepted in the context, was considered to enable relactation achievement in one of the papers (Agarwal & Jain, 2010). Mehta et al. (2018) found no statistical association between relactation and the use of lactogogues, although one case study suggested that those could have had a positive psychological effect besides stimulating lactation (Muresan, 2011). Family and husband support during lactation was reported as an enabling factor to achieve relactation in several studies (Abul‐Fadl et al., 2012; De et al., 2002; Mehta et al., 2018; Muresan, 2011). The provision of intensive support by lactation specialised staff was also considered a key feature (De et al., 2002; Gallardo, 2017; Kayhan‐Tetik et al., 2013). One study tested different educational models to encourage relactation. Among the mothers who relactated, the majority had been counselled following the coaching approach (50%), and the problem‐solving approach (45%). The cautionary or risk approach was the least effective way to provide relactation support (Abul‐Fadl et al., 2012).

**Figure 2:**
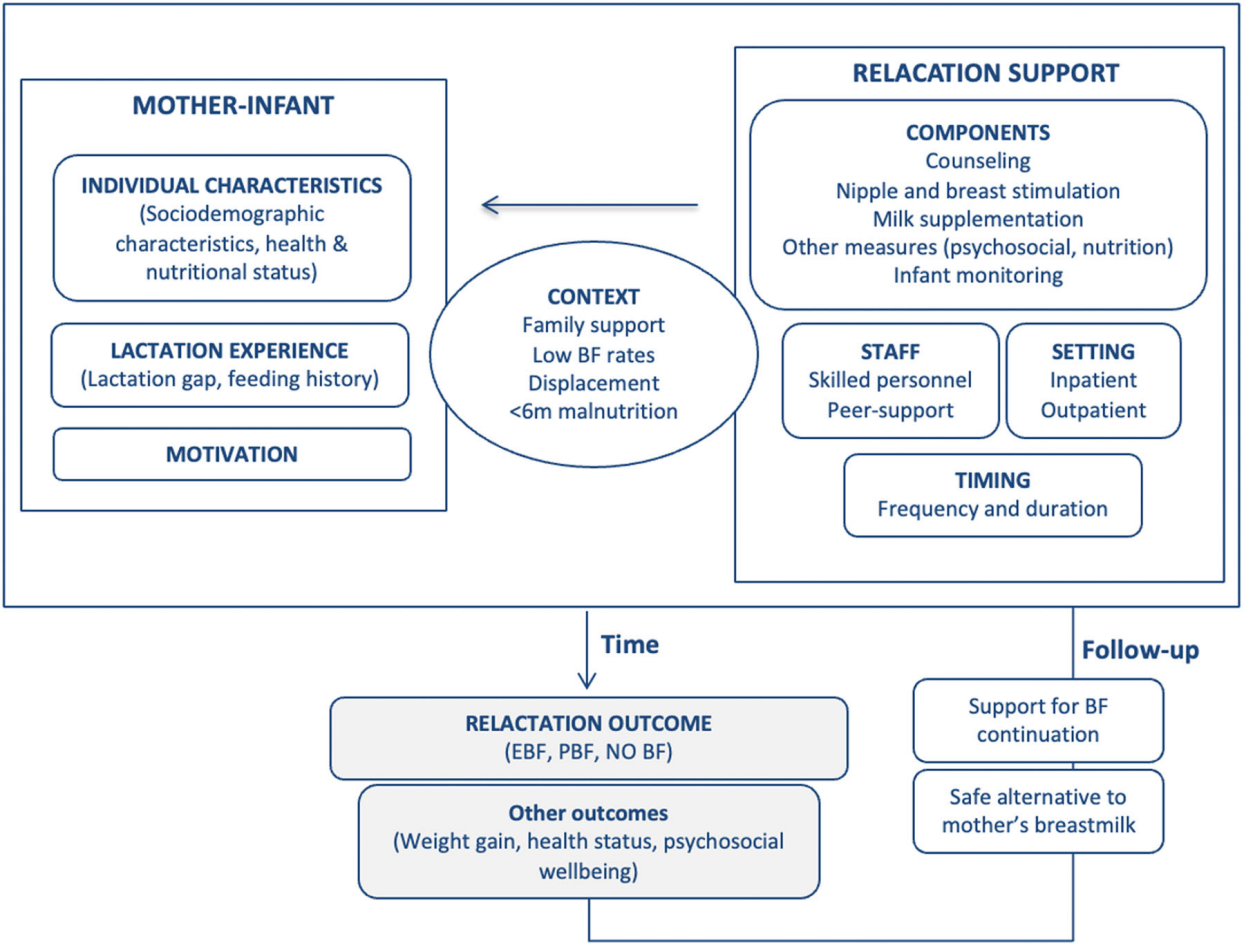
Factors involved in the implementation and outcomes of relactation support interventions

The results of this review served to develop a conceptual model encompassing the main components and associated factors of relactation support interventions (Figure 2).

## 4 DISCUSSION

This systematic review identified 16 studies describing the characteristics and outcomes of relactation interventions targeting biological mothers who were not BF their infants <6 m. The interventions were most frequently implemented during infants' hospital admission and applied the main WHO essential measures for relactation. In most studies, a high proportion of mothers re‐established BF after relactation support, although less than half achieved exclusive BF. The findings gathered through this review go in line with the experiences documented in the WHO guidelines (WHO, 1998). Yet this study adds operational perspectives to complement previous knowledge and recommendations.

This review confirms that motivated mothers with younger infants and short lactation gap, who are supported by their families are the best placed to achieve relactation. In other cases, relactation is also possible but requires longer and more intensive involvement. In contexts where exclusive BF rates are low or formula consumption is common, relactation support will probably be less accepted and more challenging (Sudfeld et al., 2012). This fact may have influenced the modest results of the reviewed interventions applied in Brazil and Egypt, (Abul‐Fadl et al., 2012; J. G. Alves et al., 1999) where national exclusive BF rates were low at the time studies were conducted (Boccolini et al., 2017; Tawfik et al., 2019). In cultures where infant care practices entail mother‐infant proximity and frequent feeding, relactation may also be more successful (Gribble, 2004).

In line with the results of this review, several authors have also shown mothers' unwillingness to try relactation or their refusal to receive inpatient support just for this purpose (Mande et al., 2017; Praborini et al., 2016; van Immerzeel et al., 2019). Mothers' wishes and contextual situations should be accounted for when developing realistic interventions that mothers can adhere to. The hospitalisation of infants seems a good opportunity to detect those who are not BF and offer their mothers relactation support during admission. This is already recommended for the treatment of infants with severe acute malnutrition (WHO, 2013), and proposed for prevention of hospital‐acquired malnutrition (J. G. B. Alves, 2006). Outpatient follow‐up after hospital discharge should be considered, since the time needed to establish lactation can exceed the days of hospitalisation (Nuzhat et al., 2019) and mothers are likely to stop or reduce BF once at home (Mahgoub et al., 2001; Nyati et al., 2014; Oberlin, 2006). Outpatient interventions are generally preferred by mothers and seem potentially effective to support relactation in certain contexts. Yet, those should include regular face‐to‐face support, as suggested by different systematic reviews looking at the effectiveness of different BF support packages (Benedict et al., 2018; McFadden et al., 2017; Rana et al., 2021). Supporting BF only through phone calls have not shown the aimed‐for effect (Rana et al., 2021).

The confidence of the health care staff in the process of relactation and its capacity to be successful was not explored in the reviewed studies, although this is likely influencing its outcomes, as suggested by Gribble (2004). The recruitment and involvement of staff with specific capacities and motivation to effectively support mothers for relactation frequently remain a challenge (Burrell et al., 2020; Lelijveld et al., 2014; Mande et al., 2017). The provision of BF training to health care staff has shown some small positive effects, although evidence available is scarce and of low quality (Gavine et al., 2017). No studies to date assess the effect of staff training on relactation outcomes. The benefits of peer support for BF have been largely discussed in the literature, (Shakya et al., 2017; Sudfeld et al., 2012) and recently revealed promising results to help relactation among hospitalised malnourished infants in Kenya (Mwangome et al., 2020).

The practicalities of breast stimulation are poorly described in available literature, despite underlined challenges (Burrell et al., 2020; Lelijveld et al., 2014; Mande et al., 2017; Muresan, 2011; Oberlin, 2006; van Immerzeel et al., 2019). In two studies reviewed, applying the supplementary suckling technique by pushing the milk with a syringe through the tube attached to the nipple, was positively valued in terms of feasibility, acceptance, and outcomes (Agarwal & Jain, 2010; Kayhan‐Tetik et al., 2013). This supplementary suckling method could be considered especially at the beginning of relactation, when infants may experience more difficulties suckling. In one study, a supplementary suckling technique was used for outpatient support in a resource constraint setting. This practice is currently discouraged as part of Infant and Young Child Feeding programmes in emergencies, due to the difficulties to ensure adequate hygiene (i.e., cleaning and maintaining tubes properly) and the consequent increased risk of infection (IFE Core Group, 2017). Although little attention was given to it, the need for milk supplementation should be considered during the relactation process, both while exclusive BF is achieved and afterwards, for mothers who are discharged partially or not BF.

Promoting maternal wellbeing should be central during relactation support as the provision of breastmilk cannot be separated from the process of BF, a complex personal experience requiring mothers' constant embodied commitment (Stearns, 2013). Initiatives like the Community Management of At‐risk Mothers and Infants under 6 months of age already advocate for the integral management of infant‐mother pairs. In line with this, they emphasise the need to assess and safeguard mothers' nutritional, physical and mental health (ENN, 2021). The potential association of maternal factors (e.g., age, stress, nutritional status, number of births and BF self‐efficacy) and the achievement of relactation should be better studied in future research.

Among the included studies, only one focused on humanitarian settings and showed poor engagement and limited success (Burrell et al., 2020). Other similar experiences reinforce the challenging nature of relactation in these settings. In a report evaluating the use of supplementary suckling technique to increase breastmilk production at therapeutic feeding units in Afghanistan, only one‐third of infants achieved exclusive BF on admission, which seemed to decrease after discharge (Oberlin, 2006). Haidar et al. (2017) also described the struggles and moral dilemmas arising when encouraging relactation among displaced mothers during the Mosul conflict in Iraq, where BF was not a common practice. Until more context‐specific knowledge is generated, the points discussed above could be applied in humanitarian settings, while reflecting upon key operational aspects like the possibility to access affected populations or the availability of resources in the different emergency phases. Low baseline BF rates and a high prevalence of malnutrition among infants <6 m will suggest a critical need for relactation support (IFE Core Group, 2017). At the same time, organisations providing emergency response should be aware that in such circumstances, relactation will not be a solution to ensure appropriate nutrition for all non‐breastfed infants. Therefore, the provision of safe alternatives to mothers' breastmilk should be carefully estimated and planned for. The current emergency response in Ukraine, where exclusive BF is low and affected populations are in transit or difficult to reach, portrays this issue (Rahimov et al., 2022).

### 4.1 Limitations and methodological considerations

Since the publication of the WHO guidelines (WHO, 1998), this is the first study systematically compiling relactation interventions and analysing in depth its features, implementation and outcomes. Nonetheless, this study was limited by the scarce number of studies available, mostly observational, of varying quality and mainly representing Asian middle‐income countries. Relactation support interventions targeting nonbiological mothers, mothers with low milk supply, or infants >6 m were not investigated, and we only searched for articles published in English and Spanish. Relactation support for wet nurses or adoptive mothers is not uncommon in humanitarian settings and should be further studied in detail. Reporting bias should be considered since some studies may have only reported successful cases but not unsuccessful ones. The way lactation failure was measured before starting relactation support was not specified in some cases, being impossible to ascertain whether some mothers were still producing milk even if not BF at that time. This unclarity in baseline measurement makes it difficult to compare studies and outcomes. Selection bias, by including only healthy and motivated mothers or hospitalised infants, could have also led to overestimating the overall positive impact of this type of intervention. Still, the knowledge derived from this review may inform practice, giving the lack of access to a higher level of evidence (Munn et al., 2020; Murad et al., 2018).

Future research should include controlled experimental studies to compare the impact of different methods of relactation support (e.g., counselling, breast stimulation or milk supplementation approaches and techniques) and focus on vulnerable populations (e.g., malnourished infants in humanitarian settings). Study design and reporting should be improved, possibly using the conceptual model presented in this study. Several outcome measurements along the follow‐up period would provide a better understanding of whether BF is sustained over time. This could also limit the possible Hawthorne effect during the intervention time—by which the sole action to participate in research can modify caregivers' behaviour, regardless of the intervention (Laborie et al., 2022). Aside from effectiveness, insights on feasibility, appropriateness and meaningfulness are also considered to be at the centre of evidence‐based health care (Jordan et al., 2019). Hence, those should be included when conducting research to inform the design of relactation support interventions.

## AUTHOR CONTRIBUTIONS

Nieves Amat Camacho, Ourania Kolokotroni and Johan von Schreeb contributed to formulating the research question and designing the study. Nieves Amat Camacho and Ourania Kolokotroni screened and discussed search results for eligibility and inclusion. Nieves Amat Camacho analysed extracted data and wrote the first draft of the manuscript. Ourania Kolokotroni, Johan von Schreeb and Francesco Della Corte revised the synthesis. All authors read and approved the final manuscript.

## CONFLICT OF INTEREST

The author declare no conflict of interest.

## Supporting information

Supporting information.

## Data Availability

Data sharing is not applicable to this article as no new data were created or analysed in this study.
