# Supplementary material for: Interventions to support the re‐establishment of breastfeeding and their application in humanitarian settings: A systematic review
Source: Matern Child Nutr. 2022 Oct 12;19(1):e13440. doi: 10.1111/mcn.13440 (PMC9749597; doi:10.1111/mcn.13440)

**APPENDICES**

**APPENDIX 1. Search strategy**

Documentation of search strategies – University Library search consultation group, Karolinska Institutet

**Medline**

| Interface: Ovid  Date of Search: 4 Sept 2020  Number of hits: 36  Comment: In Ovid, two or more words are automatically searched as phrases; i.e. no quotation marks are needed | Field labels   - exp/ = exploded MeSH term - / = non exploded MeSH term - .ti,ab,kf. = title, abstract and author keywords - adjx = adjacent within x words, regardless of order - * = truncation of word for alternate endings |
| --- | --- |
| 1. (relactat* or re-lactat*).ti,ab,kf. 2. Limit to (yr="1998 -Current" and (english or spanish)) | |

**Embase**

| Interface: embase.com  Date of Search: 4 Sept 2020  Number of hits: 43  Comment: Emtree is the controlled vocabulary in Embase | Field labels   - /exp = exploded Emtree term - /de = non exploded Emtree term - ti,ab = title and abstract - NEAR/x = adjacent within x words, regardless of order - * = truncation of word for alternate endings |
| --- | --- |
| 1. (relactat*:ti,ab,kw OR 're lactat*':ti,ab,kw) 2. [1998-2020]/py AND ([english]/lim OR [spanish]/lim) | |

**PubMed Central**

| Interface: Wiley  Date of Search: 4 Sept 2020  Number of hits: 61 | Field labels   - * = truncation of word for alternate endings |
| --- | --- |
| 1. (relactat* or re-lactat*) 2. Publication date from 1998/01/01 to 2020/12/31 | |

**Web of Science Core Collection**

| Interface: Clarivate Analytics  Date of Search: 4 Sept 2020  Number of hits: 47 | Field labels   - TS/Topic = title, abstract, author keywords and Keywords Plus - NEAR/x = adjacent within x words, regardless of order - * = truncation of word for alternate endings |
| --- | --- |
| 1. TOPIC:  ((relactat* or re-lactat*) ) 2. Refined by: LANGUAGES: ( ENGLISH OR SPANISH ) Timespan=1998-2020 | |

**Global Health**

| Interface: Ovid  Date of Search: 4 Sept 2020  Number of hits: 26  Comment: In Ovid, two or more words are automatically searched as phrases; i.e. no quotation marks are needed | Field labels   - exp/ = exploded controlled term - / = non exploded controlled term - .ti,ab,id. = title, abstract and author keywords - adjx = adjacent within x words, regardless of order - * = truncation of word for alternate endings |
| --- | --- |
| 1. (relactat* or re-lactat*).ti,ab,id. 2. Filter: years 1998-2020, Languages: English & Spanish | |

**Cinahl**

| Interface: Ebsco  Date of Search: 4 Sept 2020  Number of hits: 19 | Field labels   - MH+ = exploded Cinahl Heading - MH = non exploded Cinahl Heading - TI = title - AB = abstract - Nx = adjacent within x words, regardless of order - * = truncation of word for alternate endings |
| --- | --- |
| 1. TI ( (relactat* or re-lactat*) ) OR AB ( (relactat* or re-lactat*) ) 2. Limiters - Published Date: 19980101-20201231.  Narrow by Language: - english & spanish | |

**APPENDIX 2. Quality assessment - Johanna Briggs Institute (JBI) Critical Appraisal Tools**

**Case series/Descriptive studies**

| **Questions** | **Burrell** | **Banapurmath** | **De** | **Gallardo** | **Tomar** |
| --- | --- | --- | --- | --- | --- |
| 1. Were there clear criteria for inclusion in the case series? | Yes | Yes | Yes | Unclear | Yes |
| 2. Was the condition measured in a standard, reliable way for all participants included in the case series? | Yes | Yes | Yes | Unclear | Unclear |
| 3. Were valid methods used for identification of the condition for all participants included in the case series? | Yes | Unclear | Yes | Unclear | Yes |
| 4. Did the case series have consecutive inclusion of participants? | Yes | Unclear | Unclear | Unclear | Unclear |
| 5. Did the case series have complete inclusion of participants? | Yes | Unclear | Unclear | Unclear | Unclear |
| 6. Was there clear reporting of the demographics of the participants in the study? | Yes | Yes | Yes | No | Yes |
| 7. Was there clear reporting of clinical information of the participants? | No | Unclear | No | No | No |
| 8. Were the outcomes or follow up results of cases clearly reported? | Unclear | Yes | Yes | Yes | Yes |
| 9. Was there clear reporting of the presenting site(s)/clinic(s) demographic information? | Yes | No | No | No | No |
| 10. Was statistical analysis appropriate? | Unclear | Unclear | Yes | Yes | Yes |
| **Overall score** | 7/10 | 4/10 | 6/10 | 2/10 | 5/10 |
| **Comments**  Considered *clinical informatio*n in Q.7 as nutritional status of the infant or clinical data if infants were sick.  Considered *condition measured* in Q.3 and Q.4 as non-breastfed infants U6M, and *outcome measured* in Q.9 as relactation achievement after the intervention  No definition of outcome  No information about definition or method for measuring outcome (i.e. 24h recall, no top milk given) | | | | | |

**Case reports**

| **Questions** | **Muresan** | **Agarwal** | **Kayhan-Tetik** | **Menon** |
| --- | --- | --- | --- | --- |
| 1. Were patient’s demographic characteristics clearly described? | Yes | No | Yes | No |
| 2. Was the patient’s history clearly described and presented as a timeline? | Yes | Yes* | Yes | Yes |
| 3. Was the current clinical condition of the patient on presentation clearly described? | Yes | Yes | Yes | Yes |
| 4. Were diagnostic tests or assessment methods and the results clearly described? | NA | NA | NA | NA |
| 5. Was the intervention(s) or treatment procedure(s) clearly described? | Yes | Yes | Yes | No |
| 6. Was the post-intervention clinical condition clearly described? | Yes | Yes | Yes | Yes |
| 7. Were adverse events (harms) or unanticipated events identified and described? | Yes | No | No | No |
| 8. Does the case report provide takeaway lessons? | Yes | Yes | Yes | Yes |
| **Overall score** | 7/7 | 5/7 | 6/7 | 4/7 |
| **Comments:**  * Information about the characteristics of the mother missing | | | | |

**Cross-sectional study**

| **Questions** | **Mehta** |
| --- | --- |
| 1. Were the criteria for inclusion in the sample clearly defined? | Yes |
| 2. Were the study subjects and the setting described in detail? | Yes |
| 3. Was the exposure measured in a valid and reliable way? | Yes |
| 4. Were objective, standard criteria used for measurement of the condition? | No |
| 5. Did the case series have complete inclusion of participants? | Yes |
| 6. Was there clear reporting of the demographics of the participants in the study? | Yes |
| 7. Was there clear reporting of clinical information of the participants? | Yes |
| 8. Was statistical analysis appropriate? | No |
| **Overall score** | 6/8 |

**Cohort studies (including uncontrolled longitudinal studies)**

| **Questions** | **Abul-Fadl** | **Nuhzat** | **Bezerra Alves** | **Cluet de Rodriguez** | **Fuenmayor** |
| --- | --- | --- | --- | --- | --- |
| 1. Were the two groups similar and recruited from the same population? | Unclear | Yes | Yes | Yes | N/A |
| 2. Were the exposures measured similarly to assign people to both exposed and unexposed groups? | Yes | Yes | Yes | Yes | N/A |
| 3. Was the exposure measured in a valid and reliable way? | Yes | Yes | Yes | Yes | Yes |
| 4. Were confounding factors identified? | No | Unclear | No | Yes | Unclear |
| 5. Were strategies to deal with confounding factors stated? | No | Unclear | No | Yes | Unclear |
| 6. Were the groups/participants free of the outcome at the start of the study (or at the moment of exposure)? | Yes | Yes | Unclear | Yes | Yes |
| 7. Were the outcomes measured in a valid and reliable way? | Yes | Yes | Yes | Yes | Yes |
| 8. Was the follow up time reported and sufficient to be long enough for outcomes to occur? | Yes | No | No | Yes | Yes |
| 9. Was follow up complete, and if not, were the reasons to loss to follow up described and explored? | Yes | Unclear | Yes | Yes | Yes |
| 10. Were strategies to address incomplete follow up utilized? | NA | Unclear | NA | NA | NA |
| 11. Was appropriate statistical analysis used? | Yes | Yes | Unclear | Yes | Yes |
| **Overall score** | 7/10 | 6/11 | 5/10 | 10/10 | 5/10 |
| **Comments** | Q1. Characteristics of the mothers-infants assigned to each of the communication models is not specified, although all randomly assigned from the same population sample | Q.1. Comparisons between EBF and NBF groups on discharge  Could also be assessed as case series | Q4-5. No adjustment for covariates  Q7. Main outcome is not relactation (hospital induced malnutrition) | Q4-5. Confounding could only be prolactin levels before relactation, but those were measured.  Q7. Main outcome is not relactation (prolactin levels) | Q1. Uncontrolled longitudinal study |

**Quasi-experimental studies**

| **Questions** | **Nyati** |
| --- | --- |
| 1. Is it clear in the study what is the ‘cause’ and what is the ‘effect’ (i.e. there is no confusion about which variable comes first)? | Yes |
| 2. Were the participants included in any comparisons similar? | Yes |
| 3. Were the participants included in any comparisons receiving similar treatment/care, other than the exposure or intervention of interest? | Yes |
| 4. Was there a control group? | No |
| 5. Were there multiple measurements of the outcome both pre and post the intervention/exposure? | Yes |
| 6. Was follow up complete and if not, were differences between groups in terms of their follow up adequately described and analyzed? | Yes |
| 7. Were the outcomes of participants included in any comparisons measured in the same way? | NA |
| 8. Were outcomes measured in a reliable way? | Yes |
| 9. Was statistical analysis appropriate? | Yes |
| **Overall score** | 7/8 |
| **Comments:**  This study followed a **single group** pre-test/post-test design  Q.5. Study measured pre and post-test. Post-test outcome measured multiple times (to assess the time to achieve relactation) | |

**Summary JBI quality assessment**


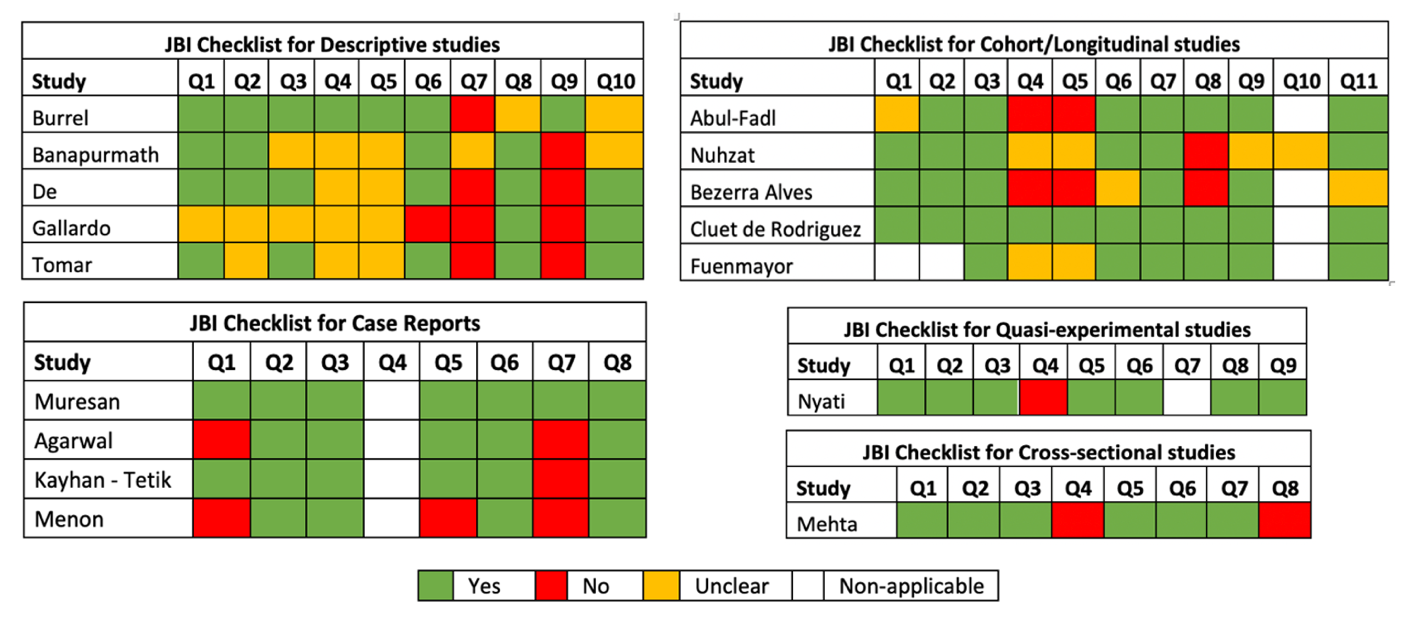

Supplement: Supplementary file 1 — Supporting information. [file MCN-19-e13440-s001.docx]
